# Ostial vs Left Main Crossover Stenting in Isolated Ostial Left Anterior Descending Stenosis: A Single-Center Retrospective Cohort Study

**DOI:** 10.1016/j.jscai.2026.105526

**Published:** 2026-08-04

**Authors:** William E. Camilleri, Eleni Ntantou, Alexandr Valentijn Kloosterman, Joost Daemen, Jeroen Wilschut, Isabella Kardys, Rutger Jan Nuis, Roberto Diletti, Karim Mahmoud, Nicolas M. Van Mieghem, Wijnand K. Den Dekker

**Affiliations:** Cardiovascular Institute, Department of Cardiology, Thoraxcenter, Erasmus MC, University Medical Center Rotterdam, the Netherlands

**Keywords:** crossover stenting, left anterior desending coronary artery, left main coronary artery, ostial stenting

## Abstract

**Background:**

Limited data exist on the optimal stenting strategy for isolated ostial left anterior descending coronary artery (LAD) lesions. The aim of this study was to compare the outcomes in patients who had a significant isolated ostial LAD stenosis treated with either left main crossover stenting (LMCS) or focal ostial LAD stenting (FO-LAD).

**Methods:**

The primary end point of the study was target lesion failure (TLF), a composite of target lesion revascularization, target vessel myocardial infarction, and cardiac death at 2 years. Secondary end points included the individual components of the primary outcome. Additional secondary outcomes included major adverse cardiovascular events, which are a composite of all-cause mortality, myocardial infarction, cerebrovascular accident, and any unplanned revascularization.

**Results:**

A total of 522 patients were included in this study, of whom 382 underwent FO-LAD stenting and 140 underwent LMCS. The primary outcome occurred in 32 patients (22.9%) in the LMCS and in 44 patients (11.5%) in the FO-LAD group at a median follow-up of 730 days (IQR, 451-730). LMCS was associated with a higher risk of TLF compared with FO-LAD stenting (adjusted HR, 1.86; 95% CI, 1.16-2.98; *P* = .01).

With regard to secondary outcomes, LMCS was associated with more major adverse cardiovascular events (31.4% vs 15.5%; adjusted HR, 2.11 [1.41-3.17]; *P* < .001) and cardiac death (17.1% vs 7.9%; adjusted HR, 2.05 [1.19-3.55]; *P* = .01]. No significant differences were observed for target lesion revascularization and target vessel myocardial infarction.

**Conclusions:**

Left main crossover stenting was associated with a higher rate of TLF at a median follow-up of 2 years when compared with FO-LAD stenting in the treatment of patients with significant isolated ostial LAD disease.

## Introduction

Ostial lesions of the left anterior descending (LAD) coronary artery are a critical target for coronary stenting due to the large myocardial territory supplied by the LAD. Optimal treatment is therefore essential to ensure favorable long-term clinical outcomes. Ostial lesions have a higher rate of in-stent restenosis and adverse events compared with nonostial lesions.^1^, ^2^, ^3^, ^4^, ^5^ In cases of isolated ostial LAD disease, defined as no angiographically significant disease in the distal left main (LM) or ostial left circumflex (LCx) coronary artery, accurate stent positioning with complete lesion coverage is paramount.

Stenting of the ostial LAD comes with many technical challenges. Conventionally, 2 stenting techniques have been adopted for the treatment of ostial LAD lesions: focal ostial LAD (FO-LAD) stenting and LM-to-LAD crossover stenting (LMCS). If not executed correctly, FO-LAD stenting can result in geographic miss, which leaves the ostium uncovered or excessive protrusion of stent struts into the LM bifurcation. If one opts for LMCS, then there is a risk of jailing the LCx ostium and inadequate LM stent expansion/apposition.^6^ It remains unknown whether LMCS is advantageous over FO-LAD stenting in isolated significant ostial LAD disease.^7^ We sought to compare the long-term outcomes in patients who had LMCS vs FO-LAD stenting.

## Materials and methods

### Study design and patient population

We conducted a single-center retrospective cohort study to compare LMCS with FO-LAD stenting in patients with isolated significant ostial LAD disease. We reviewed all patients who underwent percutaneous coronary intervention (PCI) of the proximal LAD at the Erasmus University Medical Center (Rotterdam, the Netherlands) between June 2017 and September 2023. Procedural records and angiographic findings were assessed to obtain data on the selected treatment strategy. Patients were eligible for the study if they were older than 18 years and had received a coronary intervention for an isolated significant ostial LAD lesion. Two cardiologists reviewed all the angiograms labeled as “ostial” and “proximal” LAD interventions (W.E.C. and E.N.). Intravascular imaging was not used to define ostial LAD lesions. All assessments were angiographically based. Significant ostial LAD stenosis was defined visually as a greater than 70% stenosis within 5 mm of the ostium of the LAD. Patients were divided into 2 groups based on their stenting strategy. The first group included patients who underwent FO-LAD stenting, and the second group included patients who underwent LMCS.

We excluded patients who had a history of coronary artery bypass grafting (CABG), concomitant LM disease defined as ≥50% stenosis, concomitant ostial LCx disease defined as ≥50% stenosis, or in-stent restenosis of a previous ostial LAD stent. The ethics committee of the Erasmus Medical Center approved the research proposal and concluded that the study was not subject to the Dutch Research on Human Subject Act (MEC-2024-0097).

### Data collection

All patient and procedural data were obtained from the hospital’s electronic medical record system (HiX software, ChipSoft B.V.) and entered into an electronic database (CLINT software, Erasmus MC). To determine if events have occurred, all medical records were screened up to a maximum follow-up of 730 days. Data on mortality were obtained from the municipal personnel record database. A written questionnaire or telephone survey was used to collect information on patients whose data were not accessible or had no follow-up on the electronic system.

#### End points and definitions

The primary end point of the study was target lesion failure (TLF), a composite of target lesion revascularization (TLR), target vessel myocardial infarction (TVMI), and cardiac death. TLR was defined as repeat intervention (surgical or percutaneous) to the treated segment including the 5-mm margin proximal and distal to the stent and also including the ostium of the LCx. TVMI was defined in accordance with the fourth universal definition of myocardial infarction as evidence of myocardial necrosis in the vascular territory of a previously treated target vessel.^8^ Cardiac death was defined as death resulting from cardiovascular causes. Undetermined cause of death, defined as a death not attributable to any other category because of the absence of any relevant source documents, was classified as cardiovascular death for end point determination.^9^ Secondary end points included the individual components of the primary outcome. Additional secondary outcomes included major adverse cardiovascular events (MACE), which are a composite of all-cause mortality, myocardial infarction (MI), cerebrovascular accident (CVA), and any unplanned revascularization.

#### Statistical analysis

Categorical data were expressed as counts and percentages, whereas continuous variables were expressed as medians (first and third quartiles). Categorical variables were tested with the use of the chi-square or Fisher-Freeman-Halton exact test if expected cell counts were less than 5. For continuous variables, comparisons were made using the Mann–Whitney *U* test. The occurrence of study end points over time was analyzed using the Kaplan–Meier (KM) method, and statistical differences between curves were assessed by the log-rank test. Censoring occurred at the last follow-up date if end point-free. For the comparison between FO-LAD stenting and LMCS, a multivariable Cox model was performed. All clinical, procedural, and angiographic variables were tested in univariable Cox regression. For multivariable Cox regression analysis, covariates were selected on the basis of historical relevance and a univariable *P* value threshold of <.10 and were entered in the multivariable model to determine their independent association with TLF, cardiac death, and MACE. The covariates used in the Cox regression model for TLF were the following: age, clinical presentation, calcification (moderate/severe), previous CVA, chronic obstructive pulmonary disease (COPD), and type of stenting (ostial/crossover) (Supplemental Table S1). Clinical presentation was stratified because it violated the proportional hazards (PH) assumption. The covariates used in the Cox regression model for cardiac death were the following: age, calcification (moderate/severe), previous MI, COPD, and type of stenting (ostial/crossover). MI was stratified because it violated the PH assumption (Supplemental Table S2). The covariates used in the Cox regression model for MACE were the following: age, diabetes mellitus, hypertension, calcification (moderate/severe), previous CVA, COPD, AF or flutter, and type of stenting (ostial/crossover) (Supplemental Table S3).

The 1 variable per 10 events rule of thumb was taken into account in the multivariable models to avoid overfitting of the models. Due to the limited number of events for other outcomes, only univariable analyses were performed. The results of Cox regression were presented as hazard ratios (HR) with 95% CIs.

As a sensitivity analysis, inverse probability of treatment weighting (IPTW) based on propensity scores was also performed to account for differences in baseline characteristics between the ostial and crossover stenting groups. Propensity scores were estimated using a logistic regression model including clinically relevant baseline clinical and lesion characteristics: sex, clinical presentation, diabetes mellitus, hypertension, prior CVA, prior PCI, prior MI, COPD, and calcification severity. Stabilized weights were used to estimate the average treatment effect. Covariate balance before and after weighting was assessed using standardized mean differences, with values <.10 considered indicative of adequate balance (Supplemental Table S4). In an additional sensitivity analysis, patients with death of unknown cause were excluded, and Cox PH analyses for cardiac death, TLF, and MACE were repeated to assess the robustness of the findings.

A 2-sided *P* < .05 was considered significant. The *P* values were not adjusted for multiple testing. Data were analyzed using both SPSS version 28.0.1.0 (IBM Corp) and R version 4.4.2 (R Foundation for Statistical Computing).

## Results

### Baseline characteristics

A total of 2838 patients underwent PCI for proximal LAD lesions at our institution between June 2017 and September 2023. Of these, 1985 patients were excluded as the lesion was not deemed to be ostial. We excluded 76 patients due to a history of CABG and 255 patients due to concomitant LM or ostial LCx disease (Supplemental Figure S1). In total, 522 patients were included in this study, 382 in the FO-LAD stenting group, and 140 in the LMCS group. Clinical presentation was similar between the 2 groups. Baseline characteristics were similar between the 2 groups and are shown in Table 1.

**Table 1:** Baseline characteristics

	Ostial stenting (n = 382)	Crossover stenting (n = 140)	*P* value
Age, y	68 (59-76)	71 (62-77)	.05
Female sex	93 (24.3%)	45 (32.1%)	.09
Presentation			.49
CCS	118/382 (30.9%)	51/140 (36.4%)	
UA	30/382 (7.9%)	9/140 (6.4%)	
NSTEMI	123/382 (32.2%)	47/140 (33.6%)	
STEMI	111/382 (29.1%)	33 /140 (23.6%)	
Smoker	74/365 (20.3%)	32/134 (23.9%)	.38
Diabetes	80/382 (20.9%)	35/140 (25.0%)	.32
Hyperlipidemia	155/365 (42.5%)	70/137 (51.1%)	.08
Hypertension	210/379 (55.4%)	84/140 (60.0%)	.35
Family history of CVD	94/361 (26.0%)	39/134 (29.1%)	.50
Medical history			
Stroke	26/382 (6.8%)	16/140 (11.4%)	.09
PCI	71/381 (18.6%)	29/140 (20.7%)	.59
MI	58/380 (15.3%)	27/140 (19.3%)	.27
AF	44/377 (11.7%)	16/137 (11.7%)	.99
COPD	16/378 (4.2%)	11/139 (7.9%)	.10
Heart failure			.36
HFpEF	333/369 (90.2%)	118/135 (87.4%)	
HFmrEF	36/369 (9.8%)	17/135 (12.6%)	

Values are median (IQR) or n (%).

AF, atrial fibrillation or flutter; CCS, chronic coronary syndrome; COPD, chronic obstructive pulmonary disease; CVD, cardiovascular disease; HFmrEF, heart failure with mid-range ejection fraction; HFpEF, heart failure with preserved ejection fraction; MI, myocardial infarction; NSTEMI, non-ST-segment elevation myocardial infarction; PCI, percutaneous coronary intervention; STEMI, ST-segment elevation myocardial infarction; UA, unstable angina.

### Procedural characteristics

Compared with the FO-LAD group, patients in the LMCS group underwent intracoronary imaging more frequently (61.4% vs 40.3%, *P* < .001), had a higher rate of postdilatation (97.1% vs 80.6%, *P* < .001), more frequent use of a 7 Fr guiding catheter (2.1% vs 0%, *P* = .02), and larger stent diameters (median, 3.5 mm [3.5-4.0] vs 3.5 mm [3.0-3.5]; *P* < .001). Patients in the LMCS group had a higher percentage of calcified lesions (38.6% vs 23.3%, *P* < .001). Procedural characteristics are shown in Table 2.

**Table 2:** Procedural characteristics.

	Ostial stenting (n = 382)	Crossover stenting (n = 140)	*P* value
French size			.02
6F	382 (100%)	137 (97.9%)	
7F	0 (0.0%)	3 (2.1%)	
Access site			.47
Femoral	8 (2.1%)	5 (3.6%)	
Radial	368 (96.3%)	134 (95.7%)	
Both	6 (1.6%)	1 (0.7%)	
Plaque modification			
IVL	7 (1.8%)	5 (3.6%)	.32
Atherectomy			.11
Orbital	6 (1.6%)	6 (4.3%)	
Rotational	11 (2.9%)	6 (4.3%)	
Intracoronary imaging			<.001
IVUS	109 (28.5%)	72 (51.4%)	
OCT	45 (11.8%)	14 (10.0%)	
FFR	58 (15.2%)	25 (17.9%)	.46
Mechanical support	22/378 (5.8%)	13/138 (9.4%)	.15
Planned	12/22 (54.5%)	9/13 (69.2%)	.39
Bail out	10/22 (45.5%)	4/13 (30.8%)	
IABP	11 (2.9%)	4 (2.9%)	>.99
ECMO	6 (1.6%)	1 (0.7%)	.68
Impella	7 (1.8%)	7 (5.0%)	.06
No. of treated lesions	1 (1-2)	1 (1-2)	.64
Dominance			.50
Right	332 (86.9%)	127 (90.7%)	
Left	42 (11.0%)	12 (8.6%)	
Codominance	8 (2.1%)	1 (0.7%)	
Calcification			<.001
None	293 (76.7%)	86 (61.4%)	
Moderate	65 (17.0%)	40 (28.6%)	
Severe	24 (6.3%)	14 (10.0%)	
Long lesion	244 (63.9%)	98 (70.0%)	.19
Predilatation	289 (75.7%)	122 (87.1%)	.004
Postdilatation	308 (80.6%)	135/139 (97.1%)	<.001
No. of stents	1 (1-2)	2 (1-2)	.01
Total stent length			.01
<25 mm	131 (34.3%)	32 (22.9%)	
≥25 mm	251 (65.7%)	108 (77.1%)	
Stent diameter	3.5 (3.0-3.5)	3.5 (3.5-4.0)	<.001
Two-stent technique	0 (0.0%)	7 (5.0%)	
POT		105 (75%)	
Kissing balloon inflation		92 (65.7%)	

Data are presented as median and Q1 to Q3, or as count and percent.

ECMO, extracorporeal membrane oxygenation; F, French; FFR, fractional flow reserve; IABP, intra-aortic balloon pump; IVL, intravascular lithotripsy; IVUS, intravascular ultrasound; LAD, left anterior descending coronary artery; LM, left main coronary artery; OCT, optical coherence tomography; POT, proximal optimization technique.

### Primary and secondary outcomes

The primary outcome occurred in 32 patients (22.9%) in the LMCS group and in 44 patients (11.5%) in the FO-LAD group at a median follow-up of 730 days (IQR, 451-730). In the univariable analysis, LMCS was associated with a higher rate of TLF (HR, 2.16 [1.37-3.41]; *P* < .001). This was still significant after multivariable analysis (HR, 1.86 [1.16-2.98]; *P* = .01) (Central Illustration, Table 3). KM curves for TLF showed a significant difference for the primary end point (*P* < .001; Figure 1A).

**Table 3:** Univariable and multivariable Cox regression models for clinical outcomes in patients undergoing crossover vs ostial LAD stenting

	Crossover stenting (n = 140)	Ostial stenting (n = 382)	Univariable		Adjusted	
			Hazard ratio (95% CI)	*P* value	Hazard ratio (95% CI)	*P* value
Primary outcome						
TLF	32 (22.9%)	44 (11.5%)	2.16 (1.37-3.41)	<.001	1.86 (1.16-2.98)	.01
Secondary outcomes						
TLR	7 (5%)	14 (3.7%)	1.50 (0.60-3.71)	.39		
TVMI	5 (3.5%)	7 (1.8%)	2.09 (0.66-6.58)	.21		
Cardiac death	24 (17.1%)	30 (7.9%)	2.35 (1.37-4.01)	.002	2.05 (1.19-3.55)	.01
MACE	44 (31.4%)	59 (15.5%)	2.25 (1.52-3.32)	<.001	2.11 (1.41-3.17)	<.001

The covariates used in the Cox regression model for cardiac death were the following: age, calcification (moderate/severe), prior myocardial infarction, chronic obstructive pulmonary disease (COPD), and type of stenting (ostial/crossover).

The covariates used in the Cox regression model for MACE were the following: age, diabetes mellitus, hypertension, calcification (moderate/severe), prior cerebrovascular accident (CVA), COPD, atrial fibrillation or flutter, and type of stenting (ostial/crossover).

The covariates used in the Cox regression model for TLF are the following: age, clinical presentation, calcification (moderate/severe), prior CVA, COPD, and type of stenting (ostial/crossover).

MACE, major adverse cardiovascular events; TLF, target lesion failure; TLR, target lesion revascularization; TVMI, target vessel myocardial infarction.

**Central Illustration:**
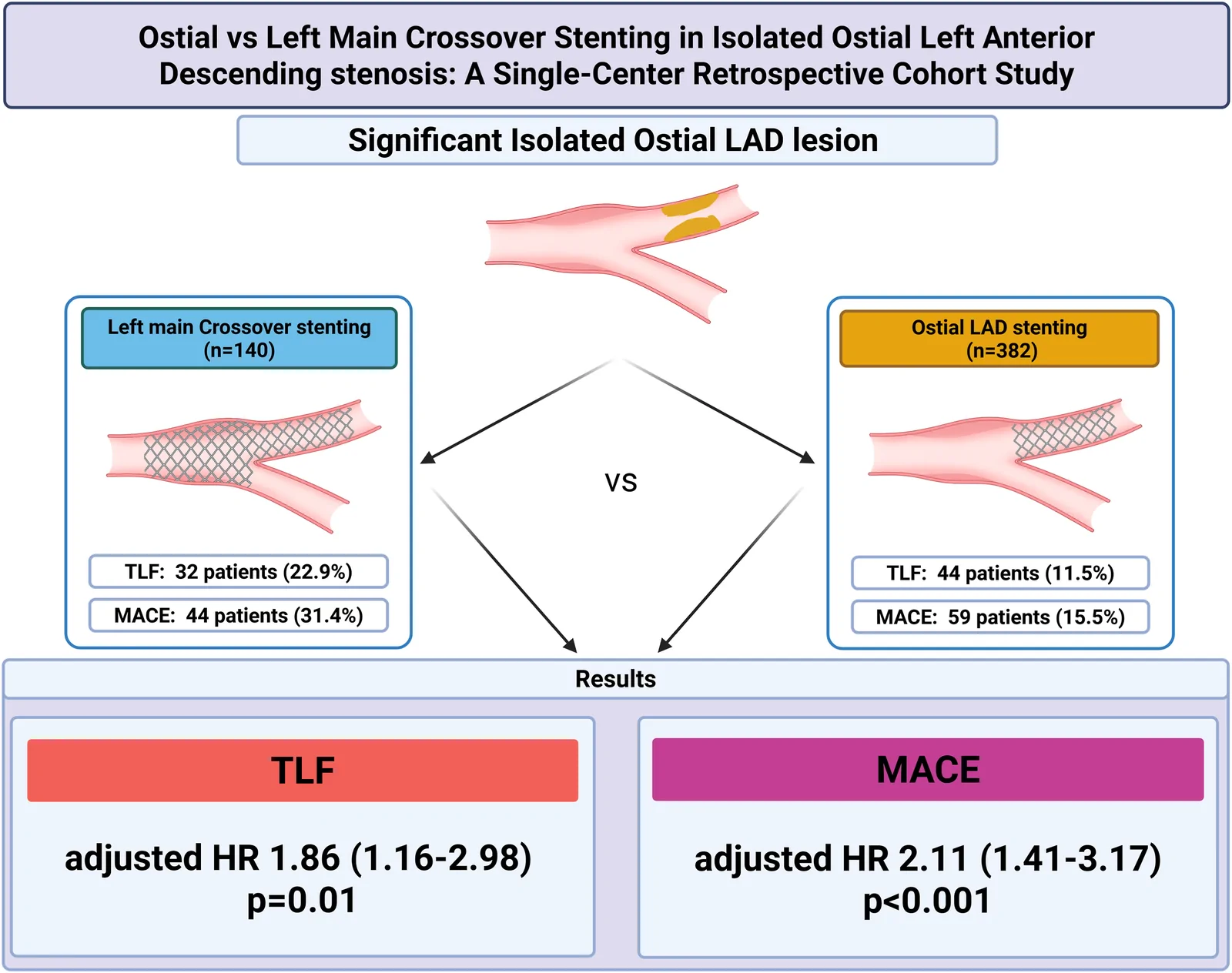
**Summary of the study findings.** LAD, left anterior descending coronary artery; MACE, major adverse cardiovascular events; TLF, target lesion failure.

**Figure 1:**
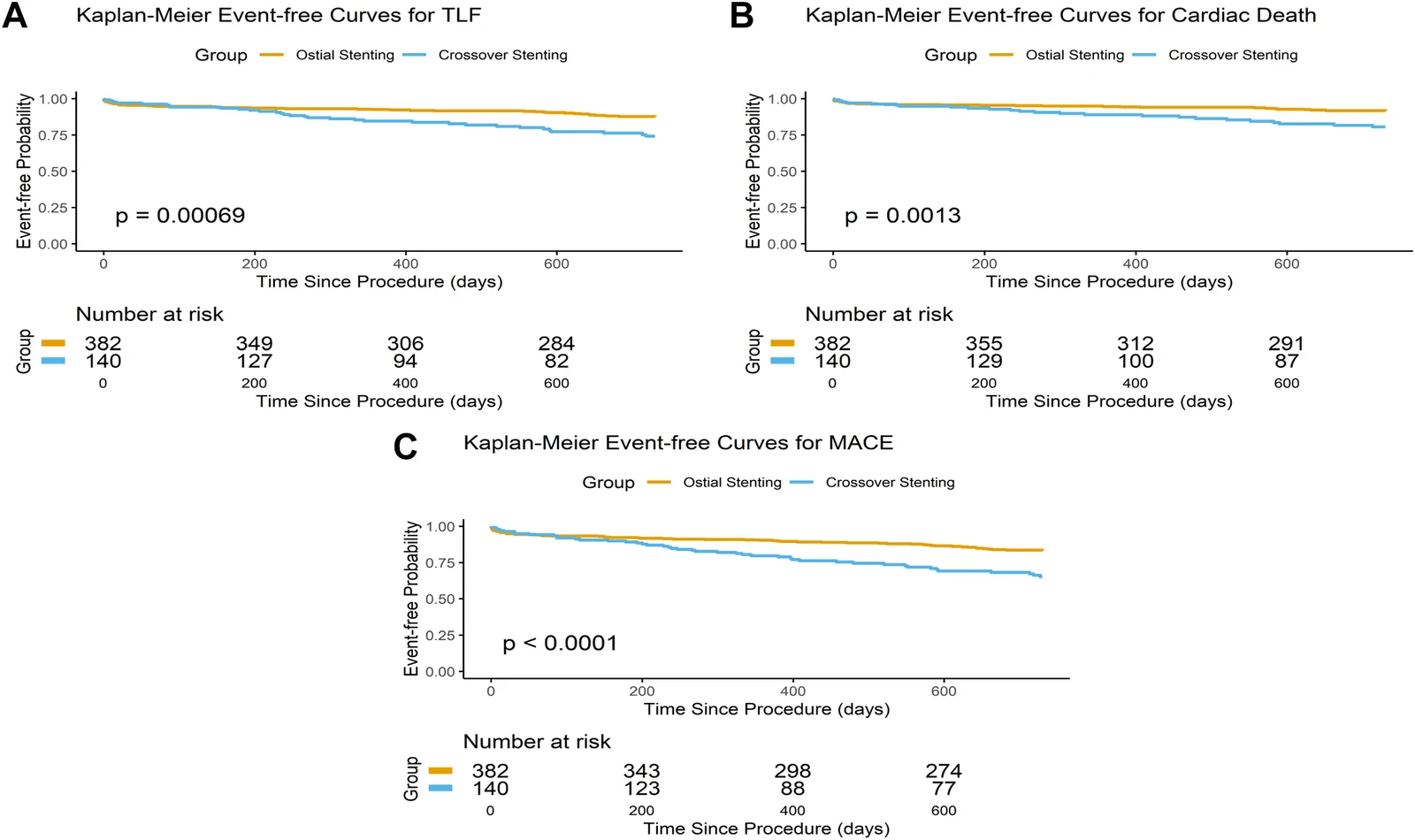
**KM curves for the primary outcome TLF (A) and the secondary outcomes cardiac death (B) and MACE (C).** KM, Kaplan–Meier; MACE, major adverse cardiovascular events; TLF, target lesion failure.

With regard to secondary outcomes, LMCS was associated with a higher rate of MACE (31.4% vs 15.5%; HR, 2.25 [1.52-3.32]; *P* < .001) and cardiac death (17.1% vs 7.9%; HR, 2.35 [1.37-4.01]; *P* = .002). Both MACE and cardiac death were still significantly higher in LMCS after multivariable analysis: HR, 2.11 (1.41-3.17); *P* < .001 and HR, 2.05 (1.19-3.55); *P* = .01, respectively (Central Illustration, Table 3). The KM curves for both cardiac death and MACE showed a significant difference (*P* = .001 and *P* < .001, respectively; Figure 1B, C).

Of the 54 cardiac deaths, 25 were attributed to a known cardiac cause. The remaining 29 deaths were of undetermined cause and were classified as cardiac deaths. In the FO-LAD group, 30 patients experienced cardiac death; of these, 16 had an unknown cause of death and 14 had a known cardiac cause of death. In the LMCS group, 24 patients experienced cardiac death; of these, 13 had an unknown cause of death and 11 had a known cardiac cause of death.

No significant differences were observed for TLR and TVMI. In the sensitivity analysis using IPTW, the LMCS group remained associated with a significantly higher risk of TLF (HR, 1.93 [1.20-3.11]; *P* = .006), cardiac death (HR, 2.07 [1.19-3.61]; *P* = .01), and MACE (HR, 2.01 [1.34-3.01]; *P* < .001). No significant associations were observed for TLR or TVMI. These findings were consistent with the primary analysis (Supplemental Table S5).

In an additional sensitivity analysis excluding patients with death of unknown cause, LMCS remained independently associated with cardiac death (adjusted HR, 2.38 [1.08-5.26]; *P* = .03), TLF (adjusted HR, 1.92 [1.06-3.48]; *P* = .03), and MACE (adjusted HR, 1.99 [1.24-3.20]; *P* = .005) (Supplemental Table S6).

The individual components of MACE are shown in Supplemental Table S7.

## Discussion

The main finding from this study was that, compared with FO-LAD stenting, LMCS was associated with a higher rate of TLF at a median follow-up of 730 days, mainly driven by cardiac death. No difference was noted for TLR and TVMI.

Importantly, the observed associations remained materially the same in a propensity score–based IPTW sensitivity analysis and in a sensitivity analysis after excluding patients with an undetermined cause of death, providing additional support for the primary findings.

Our study is the largest single-center study specifically reporting on long-term outcomes for isolated ostial LAD lesions treated with 2 different stent strategies.

The optimal revascularization strategy of isolated ostial LAD lesions has not been established.^10^ Recently, 2 meta-analyses have been published and have shown conflicting results. Desai et al^11^ showed that LMCS is associated with lower long-term risk of TLR when compared with FO-LAD stenting. On the contrary, Khairy et al^12^ showed that LMCS has similar efficacy in clinical and angiographic outcomes when compared with FO-LAD stenting. Despite including largely overlapping studies, the 2 meta-analyses by Khairy et al and Desai et al^11^ yielded divergent conclusions, particularly regarding TLR and stent thrombosis (ST). These discrepancies are primarily attributed to methodological differences. Although Khairy et al used odds ratios (ORs) and treated the data set as a single pool, Desai et al^11^ employed relative risk (RR) estimates. This matters because OR overestimates effect size when event rates are common and may be less intuitive than RR. Additionally, Khairy et al included 2 studies (Güner and Sung^13^^,^^14^) that were excluded in Desai’s^11^ analysis, potentially influencing pooled estimates. Variability in outcome definitions, weighting, and statistical handling of heterogeneity (eg, use of Galbraith plots in Khairy) further contributes to the observed differences.

Similar to a small study done by Soylu et al,^15^ we showed that LMCS had a significantly higher rate of MACE when compared with FO-LAD stenting. There are various similarities between the 2 studies including the fact that in the LMCS group, the number of stents and the stent length used were significantly higher than those in the FO-LAD group. A possible reason for this could be the large difference in luminal diameter between LM and LAD, which does not always permit the use of a single stent, but rather 2 stents in overlap in order to accommodate the discrepancy in lumen diameter between the 2 vessels. This means a greater metal load, which is a known independent predictor of restenosis.^16^^,^^17^

Our analysis demonstrated an association between LMCS and a higher incidence of TLF, with cardiac death contributing substantially to this difference. This observation may be attributable to the older age of the LMCS group and the catastrophic nature of possible LM ST, which frequently results in extensive myocardial infarction. Given the substantial myocardial territory supplied by the LM, such events often lead to sudden out-of-hospital death, precluding timely medical intervention. Interestingly, POT was not performed in 35 of 140 patients in the LMCS group. This could theoretically lead to a higher chance of stent malapposition in the LM and therefore a higher chance of fatal ST.

However, these hypotheses remain speculative, as the retrospective design of the study precludes determination of the exact mechanisms underlying the observed mortality differences. Furthermore, a substantial proportion of deaths was of undetermined cause and was classified as cardiac deaths according to ARC-2 definitions. Although sensitivity analyses yielded results consistent with the primary analysis, the mortality findings should be interpreted with appropriate caution.

FO-LAD stenting is technically demanding and if not executed correctly may result in proximal geographical miss leading to a large plaque burden (>50%) at the proximal stent edge with a high risk of plaque shifting, elastic recoil, and restenosis.^18^, ^19^, ^20^ Various techniques have been developed in order to accurately cover the ostium and achieve precise ostial stent placement. Some techniques include the stent pull-back technique, the marker wire technique, and the Szabo technique.^21^ Briefly, the latter technique involves placing a stop wire across the last strut of the stent, which will facilitate holding the proximal part of the stent right at the exact ostium of the vessel allowing accurate ostium coverage without excessive stent protrusion. However, this technique is somewhat complex with multiple steps and a steep learning curve.

The floating stent technique represents a viable strategy for the treatment of isolated ostial lesions of the LAD. This method entails deliberate deployment of a stent at the LAD ostium with minimal protrusion—typically ≤3 mm—into the LM, ensuring optimal coverage of the LAD origin while avoiding the complexity and risks associated with full left main stenting. This approach offers several advantages. It preserves the integrity of disease-free LM segments by minimizing unnecessary metal implantation, eliminating the need for aggressive postdilation within the LM, and reducing the risk of barotrauma or compromise to the LCx ostium. Medina et al^22^ demonstrated favorable medium-term outcomes using the floating stent technique for LAD ostial lesions, whereas Wu et al^23^ characterized it as a technically simple and procedurally safe compromise between ostial precision and full LM stenting. Furthermore, Kang et al^24^ showed that stent protrusion into the LM up to 3 mm did not correlate with adverse short-term angiographic or clinical outcomes.

However, it is important to recognize that the absence of early complications does not preclude longer-term or procedural risks. Protruding stent struts within the LM can serve as a nidus for thrombus formation, potentially increasing the risk of late ST.^25^ Furthermore, several studies have shown that an increased number of struts across the LCx ostium correlates with a higher incidence of subsequent LCx ostial stenosis.^26^ If the floating stent technique is not executed with precision—particularly in cases of excessive protrusion—it may also complicate future LCx interventions by hindering wire recrossing and limiting device deliverability, which can negatively impact procedural success and clinical outcomes.^27^ Given these challenges, there is a clear need for the development of dedicated devices designed specifically for non-aorto-ostial coronary lesions. Such tools could improve reproducibility, enhance precision in ostial coverage, and obviate the need for technically complex procedures—filling a current gap in the interventional cardiology toolkit.

To overcome the issues of geographical miss, LMCS has been proposed as an alternative to FO-LAD stenting.^28^^,^^29^ Ostial LAD disease is rarely isolated and often co-exists with distal LM disease.^30^ In 1 study, the extension of plaque into the distal LM was observed in 85.7% using intravascular ultrasound guidance even when angiography suggested an isolated ostial lesion.^7^ Inherent drawbacks of LMCS include stent malapposition and inadequate stent expansion in the LM^19^ and a higher incidence of carinal shift and unintended intervention to the LCx ostium, resulting in bailout PCI to the LCx. This, in turn, has implications for procedure complexity and time.^18^

An intriguing consideration in LMCS is the psychological factor influencing the decision-making process. The perceived risk of restenosis, along with the potential clinical and hemodynamic consequences, may raise concerns among operators. As a result, they may choose to avoid LMCS, aiming to reduce the risk of compromising this critical region, and instead favor an FO-LAD stenting approach. This is clearly seen from all the retrospective studies published on isolated ostial LAD stenting in which the number of FO-LAD stenting is always considerably higher than the LMCS, showing that operators opt for the more conservative ostial stenting technique to presumably prevent the complexity of LM stenting.

### Limitations

The limitations are related to the retrospective design of this study. It should be acknowledged that the LMCS group was characterized by higher lesion complexity, with more frequent calcification, greater total stent length, and more extensive use of intracoronary imaging. These procedural characteristics are recognized markers of higher risk PCI and may have influenced outcomes. Although multivariable analysis was performed to adjust for these baseline differences, residual confounding cannot be entirely excluded. Therefore, selection bias related to lesion complexity may represent a key factor contributing to the observed differences between groups. In addition, interpretation of the mortality findings is limited by the retrospective nature of the study and the substantial proportion of deaths with an undetermined cause, which were classified as cardiac deaths according to ARC-2 definitions. Another limitation is that the study relied exclusively on angiographic assessment. Consequently, there was no formal definition of focal ostial stenting beyond the operator’s intention to position the stent at the ostium of the LAD. Lastly, we could not ascertain the prevalence of subclinical LM/LCx disease, which may lead to under-recognition of plaque burden at the LAD ostium or LCx/LM edges.

## Conclusion

Left main crossover stenting was associated with higher rates of TLF at a median follow-up of 2 years when compared with focal ostial LAD stenting in the treatment of patients with significant isolated ostial LAD disease. Further prospective and randomized studies with a large sample size are warranted to confirm our findings.

## CRediT authorship contribution statement

**William E. Camilleri:** Writing – original draft, Formal analysis, Data curation, Conceptualization. **Eleni Ntantou:** Writing – review & editing, Formal analysis, Conceptualization, Data curation, Methodology, Writing – original draft. **Alexandr Valentijn Kloosterman:** Data curation. **Joost Daemen:** Writing – review & editing. **Jeroen Wilschut:** Writing – review & editing. **Isabella Kardys:** Writing – review & editing, Methodology. **Rutger Jan Nuis:** Writing – review & editing. **Roberto Diletti:** Writing – review & editing. **Karim Mahmoud:** Writing – review & editing. **Nicolas M. Van Mieghem:** Writing – review & editing. **Wijnand K. Den Dekker:** Writing – review & editing, Writing – original draft, Conceptualization.

## Declaration of competing interest

Roberto Diletti has received institutional research grants from Biotronik, Medtronic, ACIST Medical Systems, and Boston Scientific. Wijnand den Dekker has received institutional research grants from Biotronik and consulting fees from Xylocor and Vesalio. Nicolas Van Mieghem has received institutional research grants from Biotronik, Abbott, Medtronic, Edwards Lifesciences, PulseCath, Abiomed, and Daiichi Sankyo; speaker fees from Abiomed and Amgen; and a travel grant from JenaValve. Rutger Nuis received research grant support from Vifor Pharma, Merill, and consulting fees from Edwards Lifesciences, Abbott, and Boston Scientific. Joost Daemen received institutional grant/research support from Abbott Vascular, Boston Scientific, ACIST Medical, Medtronic, Microport, Pie Medical, and ReCor Medical, and consultancy and speaker fees from Abbott Vascular, Abiomed, ACIST Medical, Boston Scientific, Cardialysis BV, CardiacBooster, Kaminari Medical, ReCor Medical, PulseCath, Pie Medical, Sanofi, Siemens Health Care, and Medtronic. William E. Camilleri, Eleni Ntantou, Alexandr Valentijn Kloosterman, Jeroen Wilschut, Isabella Kardys, and Karim Mahmoud reported no financial interests.

## Footnotes

To access the supplemental material accompanying this article, visit the online version of the *Journal of the Society for Cardiovascular Angiography & Interventions* at 10.1016/j.jscai.2026.105526.
