# Supplementary material for: Ostial vs Left Main Crossover Stenting in Isolated Ostial Left Anterior Descending Stenosis: A Single-Center Retrospective Cohort Study
Source: J Soc Cardiovasc Angiogr Interv. 2026 Aug 4;5(10):105526. doi: 10.1016/j.jscai.2026.105526 (PMC13700043; doi:10.1016/j.jscai.2026.105526)
Supplement: Supplementary file 1 [file mmc1.docx]

**Supplemental Material**

| **Supplemental Table S1. Univariable Cox Regression Analysis** | | |
| --- | --- | --- |
| **Target Lesion Failure** | | |
|  | HR (95% CI) | P-Value |
| Age | 1.02 (1.00-1.04) | 0.09 |
| NSTEMI at Presentation | 2.18 (1.20-3.96) | 0.01 |
| Calcification | 1.98 (1.25-3.12) | 0.004 |
| Prior cerebrovascular accident | 1.98 (1.04-3.75) | 0.04 |
| Chronic obstructive pulmonary disease | 2.61 (1.30-5.24) | 0.007 |
| Crossover stenting | 2.16 (1.37-3.41) | <0.001 |

| **Supplemental Table S2. Univariable Cox Regression Analysis** | | |
| --- | --- | --- |
| **Cardiac Death** | | |
|  | HR (95% CI) | P-Value |
| Age | 1.03 (1.01-1.06) | 0.02 |
| Calcification | 2.10 (1.22-3.60) | 0.007 |
| Chronic obstructive pulmonary disease | 2.85 (1.29-6.31) | 0.01 |
| Prior myocardial infarction | 1.84 (1.00-3.39) | 0.05 |
| Crossover stenting | 2.35 (1.37-4.01) | 0.002 |

| **Supplemental Table S3. Univariable Cox Regression Analysis** | | |
| --- | --- | --- |
| **MACE** | | |
|  | HR (95% CI) | P-Value |
| Age | 1.02 (1.01-1.04) | 0.008 |
| Diabetes mellitus | 1.50 (0.98-2.29) | 0.06 |
| Hypertension | 1.42 (0.95-2.14) | 0.09 |
| Calcification | 1.97 (1.33-2.92) | <0.001 |
| Prior cerebrovascular accident | 2.01 (1.17-3.48) | 0.01 |
| Chronic obstructive pulmonary disease | 2.07 (1.08-3.98) | 0.03 |
| Atrial fibrillation or flutter | 1.61 (0.97-2.69) | 0.07 |
| Crossover stenting | 2.25 (1.52-3.32) | <0.001 |

**SENSITIVITY ANALYSES**

**Supplemental Table S4. Covariate Balance Before and After IPTW**

| **Variable** | **Unadjusted SMD** | **IPTW-adjusted SMD** |
| --- | --- | --- |
| Propensity score | 0.4263 | 0.0157 |
| Male sex | -0.0784 | 0.0012 |
| Presentation: CCS | 0.0522 | 0.0121 |
| Presentation: UA | -0.0153 | 0.0089 |
| Presentation: NSTEMI | 0.0155 | -0.0095 |
| Presentation: STEMI | -0.0525 | -0.0115 |
| Diabetes | 0.0385 | 0.0043 |
| Hypertension | 0.0425 | 0.0040 |
| Prior CVA | 0.0484 | 0.0032 |
| Prior PCI | 0.0193 | 0.0057 |
| Prior MI | 0.0422 | 0.0067 |
| COPD | 0.0365 | -0.0015 |
| Calcification: Moderate | 0.1144 | -0.0007 |
| Calcification: Severe | 0.0367 | 0.0038 |
| Calcification: None | -0.1512 | -0.0031 |

SMD values <0.10 were considered indicative of adequate covariate balance.

CCS, chronic coronary syndrome; COPD, chronic obstructive pulmonary disease; CVA, cerebrovascular accident; IPTW, inverse probability of treatment weighting; MI, myocardial infarction; NSTEMI, non-ST-segment elevation myocardial infarction; PCI, percutaneous coronary intervention; SMD, standardized mean difference; STEMI, ST-segment elevation myocardial infarction.

**Supplemental Table S5. IPTW Sensitivity Analysis Results**

| **Outcome** | **HR** | **95% CI** | **P-Value** |
| --- | --- | --- | --- |
| TLF | 1.93 | 1.20–3.11 | 0.006 |
| TLR | 1.44 | 0.55–3.80 | 0.46 |
| TVMI | 2.24 | 0.65–7.75 | 0.20 |
| Cardiac Death | 2.07 | 1.19–3.61 | 0.01 |
| MACE | 2.01 | 1.34–3.01 | <0.001 |

CI, confidence interval; HR, hazard ratio; IPTW, inverse probability of treatment weighting; MACE, major adverse cardiovascular events; TLF, target lesion failure; TLR, target lesion revascularization; TVMI, target vessel myocardial infarction.

**Supplemental Table S6. Sensitivity Analysis Excluding Deaths of Unknown Cause**

| **Outcome** | **Unadjusted HR (95% CI)** | **P-value** | **Adjusted HR (95% CI)** | **P-Value** |
| --- | --- | --- | --- | --- |
| Cardiac Death | 2.29 (1.04–5.06) | 0.039 | 2.38 (1.08–5.26) | 0.03 |
| TLF | 2.03 (1.14–3.64) | 0.017 | 1.92 (1.06–3.48) | 0.03 |
| MACE | 2.21 (1.39–3.50) | <0.001 | 1.99 (1.24–3.20) | 0.005 |

CI, confidence interval; HR, hazard ratio; MACE, major adverse cardiovascular events; TLF, target lesion failure.

**Supplemental Table S7. Individual Components of MACE in Patients Undergoing Left Main Crossover Stenting Versus Ostial Stenting**

|  | **Crossover Stenting** | **Ostial Stenting** |
| --- | --- | --- |
| All-cause mortality | 28 (20.0%) | 33 (8.6%) |
| Cerebrovascular accident | 3 (2.1%) | 6 (1.6%) |
| Repeat revascularization | 12 (8.6%) | 23 (6.0%) |
| Myocardial infarction | 7 (5.0%) | 12 (3.1%) |

**Supplemental Figure S1. Study flowchart**

CABG, coronary artery bypass grafting; LAD, left anterior descending coronary artery; LCx, left circumflex coronary artery; PCI, percutaneous coronary intervention.
